# An electrochemical biosensor for the rapid genetic identification of Musang King durian

**DOI:** 10.1038/s41598-022-20998-8

**Published:** 2022-11-11

**Authors:** Mohammad Malek Faizal Azizi, Sohana Romeli, Hazana Razali, Eda Yuhana Ariffin, Muhammad Afiq Tajol Ariffin, Lee Yook Heng, Norliza Abu-Bakar, Han Yih Lau

**Affiliations:** 1 Biotechnology and Nanotechnology Research Centre, Malaysian Agricultural Research and Development Institute (MARDI), Persiaran MARDI-UPM, 43400 Serdang, Selangor Malaysia; 2 Department of Chemical Sciences, Faculty of Science and Technology, Universiti Kebangsaan Malaysia, 43600 Bangi, Selangor Malaysia; 3 Horticulture Research Centre, Malaysian Agricultural Research and Development Institute (MARDI), 06050 Bukit Kayu Hitam, Kedah Malaysia

**Keywords:** Biotechnology, Chemistry

## Abstract

More than 200 different cultivars of durian exist worldwide but *Durio zibethinus* or Musang King (MK) is the most premium and prized durian fruit among the recommended varieties. Early identification of this premium variety is critical to protect from non-authentic MK durian cultivars. However, the MK variety's morphological traits are nearly identical to other varieties. Currently, the identification of durian varieties is mostly performed via evaluation of leaf shape, fruit shape, aroma, taste and seed shape and this requires trained personnel for the morphology observation. To enable the rapid identification of the MK variety, PCR amplification of ten durian varieties using six gene candidates from the chloroplast genome was first performed to obtain DNA probes that were specific to the MK durian variety. PCR amplification of ten durian varieties using primers designed confirmed that the *nadhA* gene sequence showed an obvious difference in the MK variety from other durian varieties. The unique sequence of MK was used as a DNA probe to develop an electrochemical biosensor for the direct identification of the MK durian variety. The electrochemical biosensor was based on the hybridization response of the immobilized DNA probe with the target DNA from the MK variety and was monitored via differential pulse voltammetry technique. Under optimal conditions, the DNA electrochemical biosensor showed a low detection limit at 10% of MK genomic DNA concentration with a wide linear calibration range of 0.05–1.5 µM (R^2^ = 0.9891) and RSD value of 3.77% (n = 3). The results of the developed DNA biosensor provide high promise for the development of portable sensors employed in the determination of MK variety in the field.

## Introduction

Durian is Southeast Asia’s popular fruit belonging to the genus Durio and the Malvaceae family, specifically the sub-family Bombacaceae. Durian is also known as the “king of fruit” for its formidable spiny husk, overpowering flavor, and unique odor, described as an onion-like, sulfury aroma with notes of sweet fruitiness and savory soup seasoning^1^. Among the 30 known species in the Durio genus, *D. zibethinus* is the most prized as a major Southeast Asian food crop. The three leading durian producing countries are Thailand, Malaysia, and Indonesia, with more than 250,000 ha cultivated in 2008^2^. Durian also has major economic value, as it has recently gained market penetration in China up to $600 million in durian imports in 2016 alone^3^.

More than 200 different cultivars of durian exist worldwide, encompassing a range of fruit textures, flavors, and aromas. Malaysia has more than 100 cultivars of durian^4^. In view of the numerous durian cultivars available, preferences for recommended varieties fetch a higher price as planting materials or as fresh produce. Musang King (MK) is one of the most premium and valuable durian varieties in Malaysia which was registered by the Department of Agriculture with registration code D197^5^. Musang King also known as ‘Raja Kunyit’ and ‘Mao Shan Wang’ has yellow flesh color with an obovoid to oblong fruit shape and a mix of a sweet, creamy and bitter taste. This variety was produced naturally by natural pollination and being clonally propagated on a large scale since its being registered in 1993^6^. In the propagation system, the assurance of durian cultivar identity is compulsory. Plant breeding techniques require the certainty of the variety's identity to avoid confusion. Durian breeding through artificial crossing requires enormous efforts and takes a long period of time. Experiences from previous breeding research in Malaysia and Thailand showed that the breeding process took up to 30 years to obtain new cultivars from one breeding generation^7^. To shorten the period of selection and to make it cost-effective for durian breeding, it is essential to have a durian varieties identification tool for progeny selection.

However, identification of MK variety is challenging due to some varieties showing similar morphological characteristics, which are difficult to distinguish and not applicable to opened durian fruits without shells in the markets. The distinctive odours of different durian cultivars, including MK have also been biochemically studied and characterized as a complex suite of odor-active compounds including sulfur volatiles, esters, alcohols, and acids^8, 9^. This, therefore, results in a greater demand for quality planting materials of specified variety. The determination of the durian identity can be ideally conducted by the integration of morphological and molecular characterizations. Currently, the identification of durian varieties is mostly performed via evaluation of leaf shape, fruit shape, aroma, taste and seed shape^7, 10^ and could only be by trained personnel. In 2018, a draft whole-genome assembly of the MK cultivar was reported in Singapore, providing a piece of useful information for durian agronomy^11^. The complete reported genome of MK may aid in the identification of cultivar-specific sequences, particularly Single nucleotide polymorphism (SNPs) associated with crucial cultivar-specific features (such as flavor, texture, and odor), and it may enable the molecular barcoding of distinct durian cultivars for rapid quality control and identification.

However, SNP marker validation entails labor-intensive and costly^12^. Furthermore, SNP genotyping analysis of one sample at a time is extremely expensive and time-consuming^13^. Moreover, SNPs are less polymorphic than other molecular markers because of their biallelic nature^13^. The other existing DNA markers technology, such as simple sequence repeats (SSR)^14–16^, inter-simple sequence repeat (ISSR)^17, 18^, and random amplified polymorphic DNA (RAPD) markers^19, 20^ have been deployed in the identification of selected durian varieties. Molecular markers are nucleotide sequences that represent variation in nucleotide sequences across individuals of a species and are located at a known position on the chromosome. DNA markers are widely utilized in practical agriculture monitoring, mostly due to their reliability, stability, reproducibility, efficiency, and cost-effectiveness. In 2018, the simple sequence repeats (SSR) marker was employed as an alternative technique to study genetic variation among Durian varieties in Malaysia owing to its co-dominant inheritance, multi-allelic nature, and high reproducibility compared to other markers^13, 16^. However, the development process of SSR marker is quite lengthy and expensive, and throughput is low due to automation and output data management limitations^21^.Though RAPD and ISSR markers are a suitable alternative to provide useful information for the identification of plant variety and cultivar, general genetic diversity assessment, population genetic structure and plant protection, however, the dominant character of these markers considers them inappropriate for DNA fingerprinting and genetic variation analysis^22^. Notably, the information derived from dominant genetic markers is less informative than that derived from co-dominant genetic markers, as codominant inheritance of markers can differentiate between homozygous and heterozygous states based on the generated alleles but not dominant inheritance of markers. Furthermore, some dominant genetic markers are known to suffer from poor reproducibility^13, 23^, raising concerns about the feasibility and reliability of using dominant genetic markers for downstream applications.

With the advent of DNA biosensor technology, the gap in durian variety identification can be bridged. Therefore, in this work, we attempted to develop an electrochemical DNA biosensor, which can be useful to differentiate and to distinguish the identities among the durian cultivars. This technique is highly sensitive, simple, and low cost for MK durian variety determination with good accuracy. Electrochemical DNA biosensors have been widely adopted in agriculture, particularly in disease diagnosis, due to their potential to give higher sensitivity, faster analysis, portability, and lower cost than traditional technologies^24^. The selection of the right immobilization method and the suitability of matrix type for DNA probe immobilization are imperative owing to producing a high-performance DNA biosensor. The selection of DNA probe is based on DNA barcoding for plants technique, wherein genes encoding a mitochondrial cytochrome oxidase (COI, *cox*I) subunit were extensively employed^25, 26^. The development of the DNA barcoding technique allows for high rapididentification of plant varieties and cultivars efficiently^13^. The chloroplast genome in plants is considered a potential candidate for barcoding genes due to conserved gene order, high copy number and simple amplification by PCR^13, 27^.

In this research, the six genes candidate from DNA barcoding for plants were deployed by PCR amplification of ten durian varieties to identify DNA sequences that are unique to the MK variety but not found in other durian varieties and to develop as DNA probes. The DNA probe specific to the MK durian variety was used further to develop an electrochemical biosensor for the direct identification of the MK durian variety. The biosensor was constructed from the carbon-paste screen-printed electrode (SPE), which was modified by depositing with gold nanoparticles (AuNPs) and acrylic microspheres (AcMPs) containing succinimide functional groups as the matrix for DNA probe immobilization. The succinimide functional group of the acrylic microspheres become a linker to immobilize aminated DNA probe via covalent bonds^28, 29^. The acrylic microspheres provide the advantages of small size and serve a large surface area to volume ratio for DNA probe immobilization on the surface, thus avoiding any barriers to the diffusion of reactants and products^30^. The overall electrode design provides a wide surface area for chemical reactions and excellent surface immobilization properties, which can significantly increase the effectiveness of the electrochemistry detection assay^31–33^. Thus, acrylic microspheres (AcMPs) based DNA biosensors have the potential to serve as a promising platform for the development of rapid, sensitive, specific, and portable diagnostic tools for DNA detection^34–36^.

## Results and discussion

### DNA probe determination of ‘Musang King’

**Figure 1:**
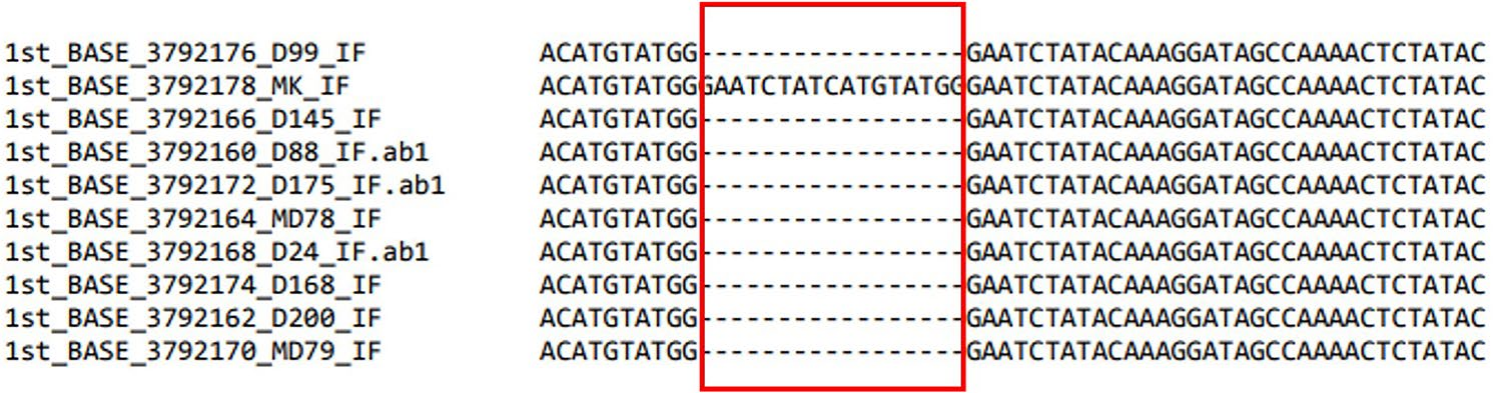
DNA sequence alignment of *nadhA* from MK, D88, D24, D99, D145, D168, D175, D200, MDUR78 and MDUR79. The red box shows the obvious comparison sequence among 10 durian varieties in which MK sequence contain unique sequence of 17 bases.

The sequencing analysis of ten durian varieties showed that there are some unique regions found in loci *nadhA* gene sequence and had obvious comparison among ten durian varieties in which D99, D145, MDUR88, D175, MDUR78, D24, D168, D200, and MDUR79 durian varieties sequences containing sequence lesion of 17 bases but not in MK DNA sequence (Fig. 1). This comparison implies that the unique sequence in the MK DNA sequence can be used as a DNA probe to differentiate between MK with the other durian varieties via electrochemical biosensor. Whereas the other five gene candidates did not show any significant differences among the ten durian varieties sequence (Fig. S1) DNA barcode candidates are based on chloroplast genome that provides some advantages such as simple and stable genetic structure, it is haploid, undergoes no (or very rare) recombination, it is generally uniparentally transmitted, and universal primers that are suitable to use for amplifying target sequences^37^. Additionally, chloroplast genomes are high conservation over nuclear and mitochondrial genomes. Therefore, partial chloroplast genome sequences are preferred and suitable to use for phylogenetic studies and species/varieties identification and discrimination with similar morphology characteristics^38–43^.

DNA barcoding has been deployed in prior studies owing to its effectiveness in plant variety and cultivar identification. The chloroplast genome is considered a promising candidate for barcoding genes in various land plants due to its advantages, such as conserved gene order, high copy number, and ease of amplification by PCR^13^. The vast of studies on plant barcoding employs one or a few plastid areas, including the protein-coding *rbcL* and matK regions, the noncoding spacer *trnH-psbA*, and *ITS* regions. For instance, the three chloroplast genomes (*ITS2* and cp gene *matK* and *rpl32-trnL* (UAG)) were successfully identified and discriminated 12 commercial varieties of kiwi fruit^44^. Likewise, Emirati date palm cultivars were able to discriminate using the chloroplast intergenic spacer *psbK-psbI*^45^. In Xinjiang, China, *Artemisia* L. varieties were identified using the chloroplast genome of *ITS* region which revealed the highest identification efficacy^46^.

### The response of the biosensor towards target DNA concentrations

**Figure 2:**
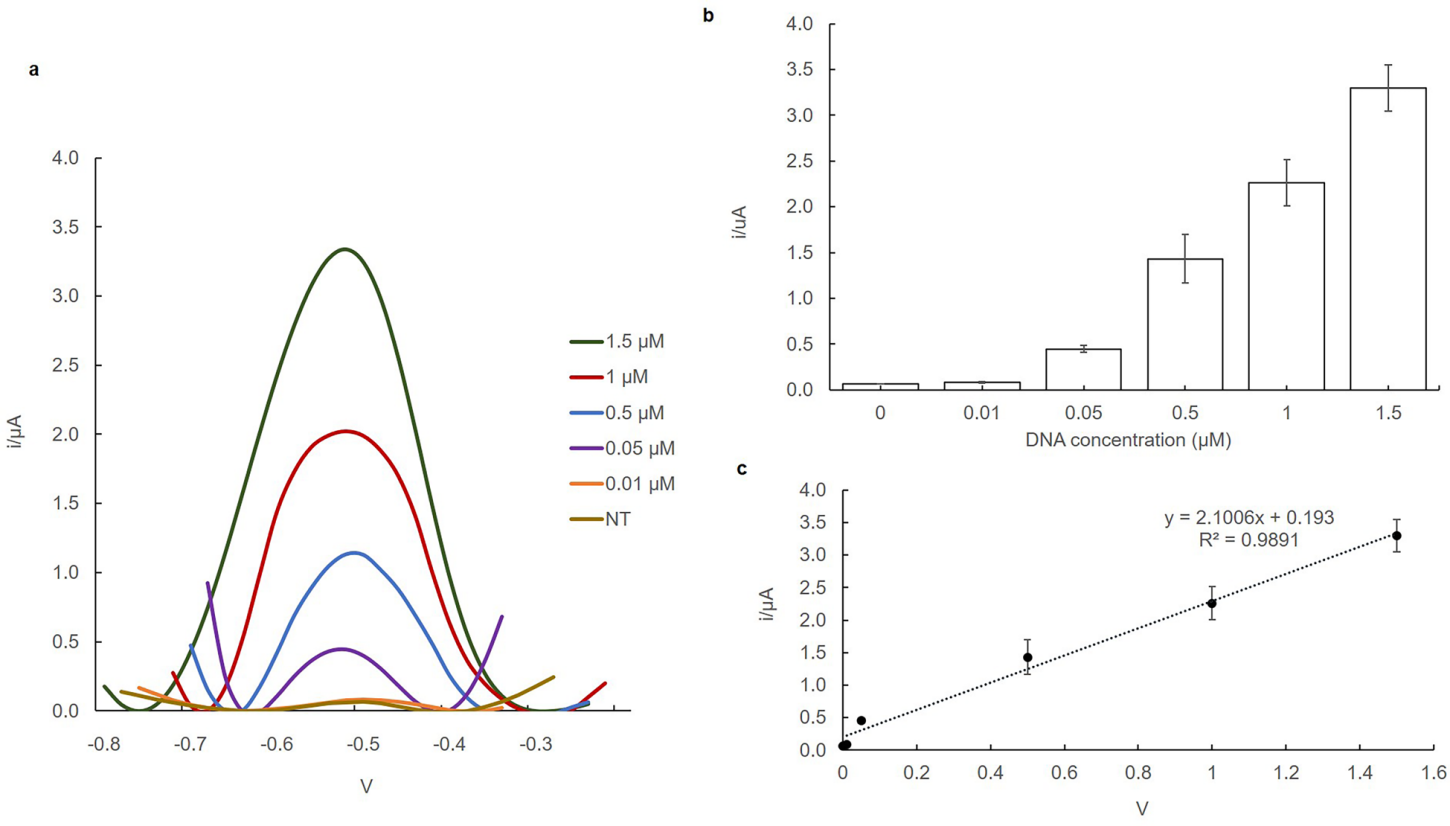
Differential pulse voltammogram signal of AcMP-AuNP-based DNA modified carbon SPEs electrode upon hybridization with different concentration of DNA target (1.5 µM, 1 µM, 0.1 µM, 0.05 µM, and 0.01 µM), and non-complementary (NC) (**a**), and the bar chart response range of the target DNA hybridized to DNA probe on acrylic microsphere (**b**). The linear response range for hybridization reaction between target DNA and DNA probe on AcMP-AuNP modified carbon SPE (**c**). The DPV peak rate was observed at − 0.55 V to − 0.45 V/s versus Ag/AgCl reference electrode in the presence of 10 mM AQMS. Error bars are standard deviations across three repetitive experiments.

Under the optimal conditions, the analytical performance of the DNA biosensor was examined using the immobilized DNA probe by the response of the AcMP-AuNP-modified carbon SPEs electrode towards an increasing concentration of synthetic target DNA (1 µM, 1.5 µM, 0.1 µM, 0.05 µM, and 0.01 µM), non-complementary (NC) DNA and no target (NT) at a scan rate of 0.04 V s^−1^, the results were illustrated in Fig. 2. The current response was increased steadily with increasing target DNA concentration at the electrode in experiment (a). The highest peak was 1.5 µM followed by 1.0 µM, 0.1 µM, 0.05 µM, 0.01 µM, and NC. The DPV peak reading of all concentrations was in the range of − 0.55 V to − 0.45 V. This indicates more DNA duplex was formed on the electrode through DNA hybridization reactions followed by anthraquninone-2-sulfonic acid monohydrate sodium salt (AQMS) intercalation. Additionally, significant current differences were observed in the DPV peak from all concentrations, implying that the DNA probes were successfully immobilized onto the AcMP via covalent bonds between succinimide group of AcMP and amine functional group of the DNA probe^28, 29^. The concentration of 1.5 µM was the highest peak, indicated hybridization where intercalation of AQMS had occurred in double-stranded DNA (dsDNA) formed in the microsphere surface with the current peak value of 3.34 µA. However, the DPV peak of NT was the lowest of other concentrations due to no DNA hybridization reaction occurring, indicating there was no specific adsorption of AQMS redox indicator onto AcMP-AuNP-based DNA modified carbon SPEs electrode^47^.

The DNA biosensor responses with five different concentrations of synthetic target DNA and no target that has been hybridized on DNA probe immobilized on AcMPs are shown in Fig. 2b. DNA biosensor response increased proportionally with increasing concentrations of synthetic DNA target immobilised. This suggests that the capacity of immobilized DNA probe to hybridize with complementary of five different synthetic target DNA concentrations has increased with an increase in DNA target attached to the AcMP-AuNP. The linear response range of the DNA biosensor from different concentrations of synthetic target DNA demonstrates that the oxidation peak current of DNA increased linearly with increasing synthetic target DNA concentration and revealed a satisfactory correlation coefficient value of R^2^ = 0.9891, and the linear equation was expressed y = 2.1006x − 0.193 with a low detection limit of 0.01 µM (Fig. 2c). The large binding surface area of the AcMPs enabled a large number of DNA molecules to bind covalently to the AcMPs via succinimide functional groups, ultimately enhance the analytical performance of the DNA biosensor in terms of dynamic linear range and detection limit^30^.

### The specificity of the biosensor response

**Figure 3:**
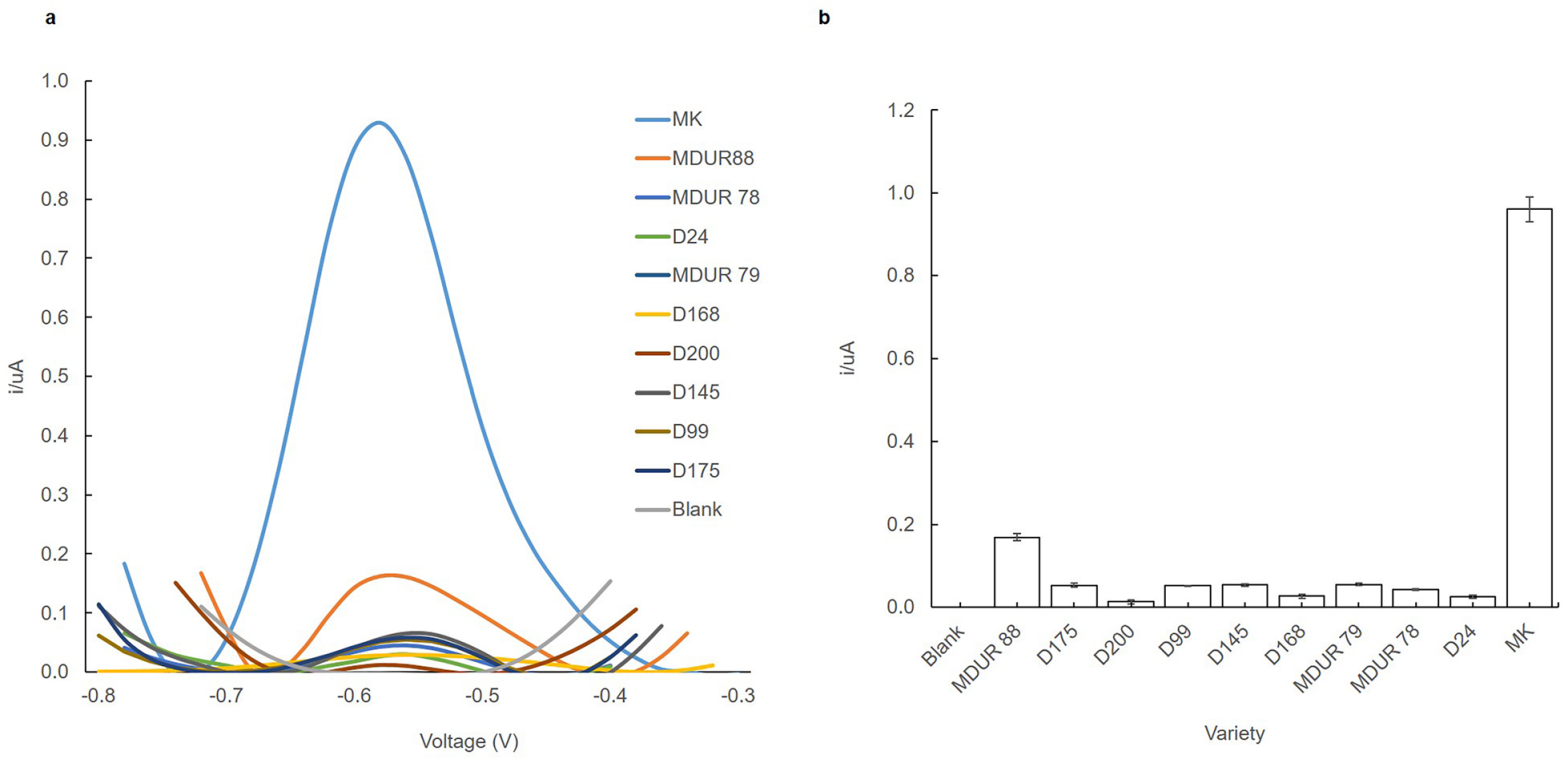
The durian varieties DNA biosensor response (**a**) and the biosensor response trends (**b**) after hybridization with different durian varieties of complementary DNA: (MK, D24, MDUR88, MDUR78, MDUR79, D168, D200, D145, D99, D175) and blank. The error bars represented standard deviations across three repetitive experiments.

Figure 3a shows the DPV response of DNA biosensors with different Durian varieties (MK, D24, MDUR88, MDUR78, MDUR79, D168, D200, D145, D99, D175) and blank (without DNA) under optimum conditions. The DPV peak near − 0.55 V indicates the oxidation of AQMS that has been intercalated into the dsDNA formed from MK DNA probe hybridized with MK complementary DNA. This result revealed that the designed DNA probe from MK is highly specific to MK. MK variety yielded the highest current response as expected and demonstrated that the immobilized MK DNA probe was selective only towards its complementary DNA as has previously reported from other studies^28, 29^. Based on Fig. 3b, MK displays the highest current value of 0.9 µA. Meanwhile, the other varieties yielded a current below 0.2 µA and near to the baseline response of the blank. This is confirmed by the absence of peak or very low current from the other Durian varieties (D24, MDUR88, MDUR78, MDUR79, D168, D200, D145, D99, D175) and blank (the absence of DNA). This indicated neither hybridization nor specific absorptions of AQMS redox indicator occurred on the electrode surface^48, 49^. Thus, it confirmed the DNA of MK has hybridized with MK DNA probe attached to the AcMP-AuNP.

### The sensitivity of the biosensor

**Figure 4:**
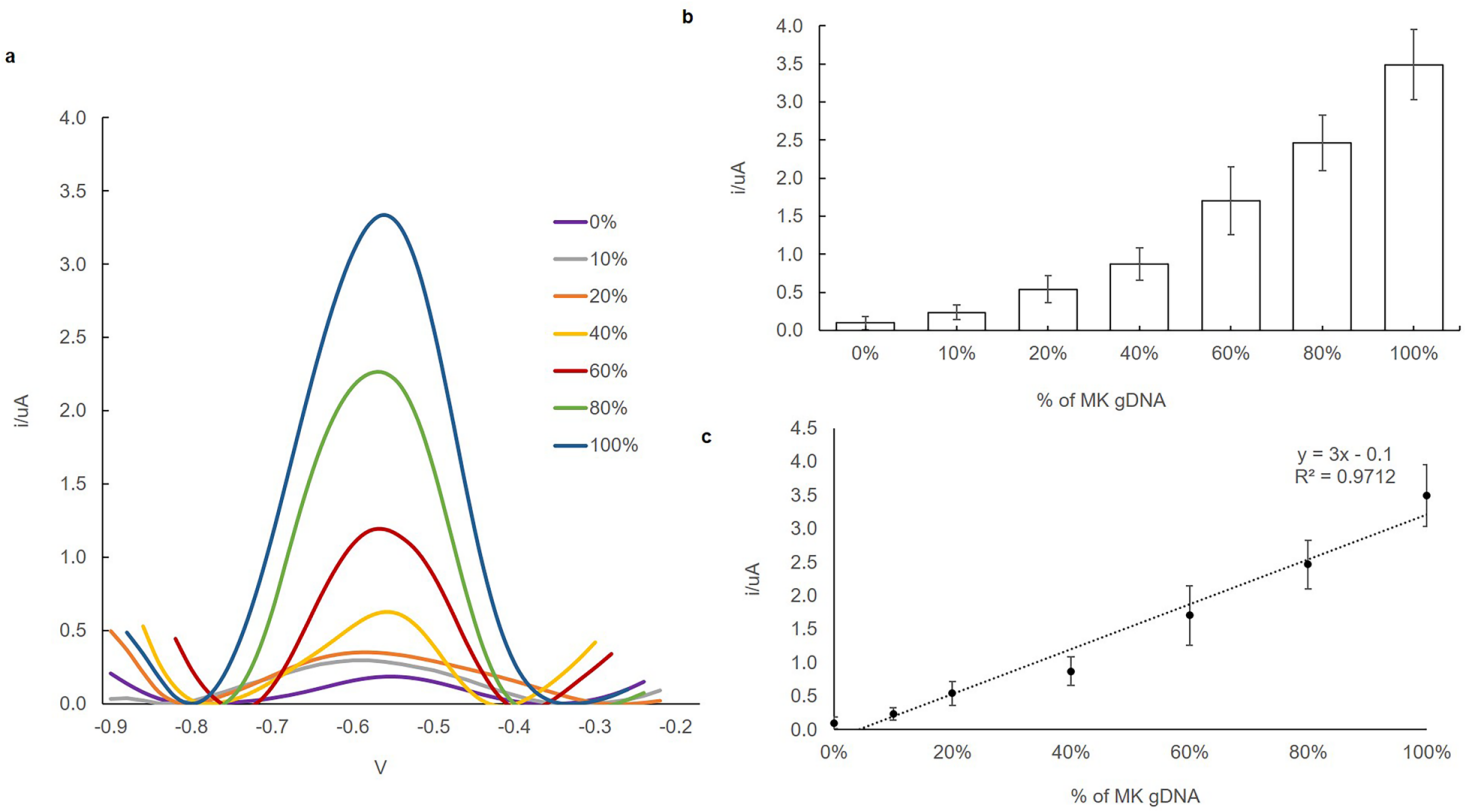
Differential pulse voltammograms (**a**) biosensor response trends; (**b**) linear response range and (**c**) of DNA probe/AuNPs/SPE electrode upon hybridisation with different concentration of MK (0%, 10%, 20%, 40%, 60%, 80%, 100%). Hybridization was performed in 0.05 M K-phosphate buffer (pH 7.0) containing 10 mM AQMS. The DPV peak rate was observed at − 0.55 to − 0.45 V/s versus Ag/AgCl reference. Error bars are standard deviations across three repetitive experiments.

To investigate the sensitivity of the biosensor, increasing amounts of MK genomic DNA were added to triplicate reactions containing a decreasing amount of MDUR88 genomic DNA. The genomic DNA mixture containing 0%, 10%, 20%, %, 40%, 60%, 80%, and 100% of MK genomic DNA. This is for the purpose of determining the biosensor response towards MK genomic DNA purity as to whether it is derived solely from clone or hybrid. Figure 4a represents the DPV of different genomic DNA concentrations of MK (0%, 10%, 20%, 40%, 60%, 80%, and 100%) in the present of MDUR88 genomic DNA. The current response of this proposed biosensor clearly increased with the increase of the target concentrations from 0 to 100%, followed by intercalation of AQMS in between the dsDNA (Fig. 4a and b). The DNA biosensor showed a good linear relationship with the percentage of MK gDNA (Fig. 4c), and the linear equation was expressed y = 3x − 0.1 (R^2^ = 0.9712) with a detection limit down to 10% of MK gDNA for sensitivity analysis. The reproducibility of the DNA biosensor was performed with 100% of MK gDNA. DNA biosensors gave satisfactory reproducibility results with 3.77%. The DPV peak reading of all concentrations was at − 0.55 V. This suggests an increase in hybridization between the immobilized DNA probes with the target DNA when the DNA concentrations increased.

### Biosensor response validation with real durian DNA samples

**Figure 5:**
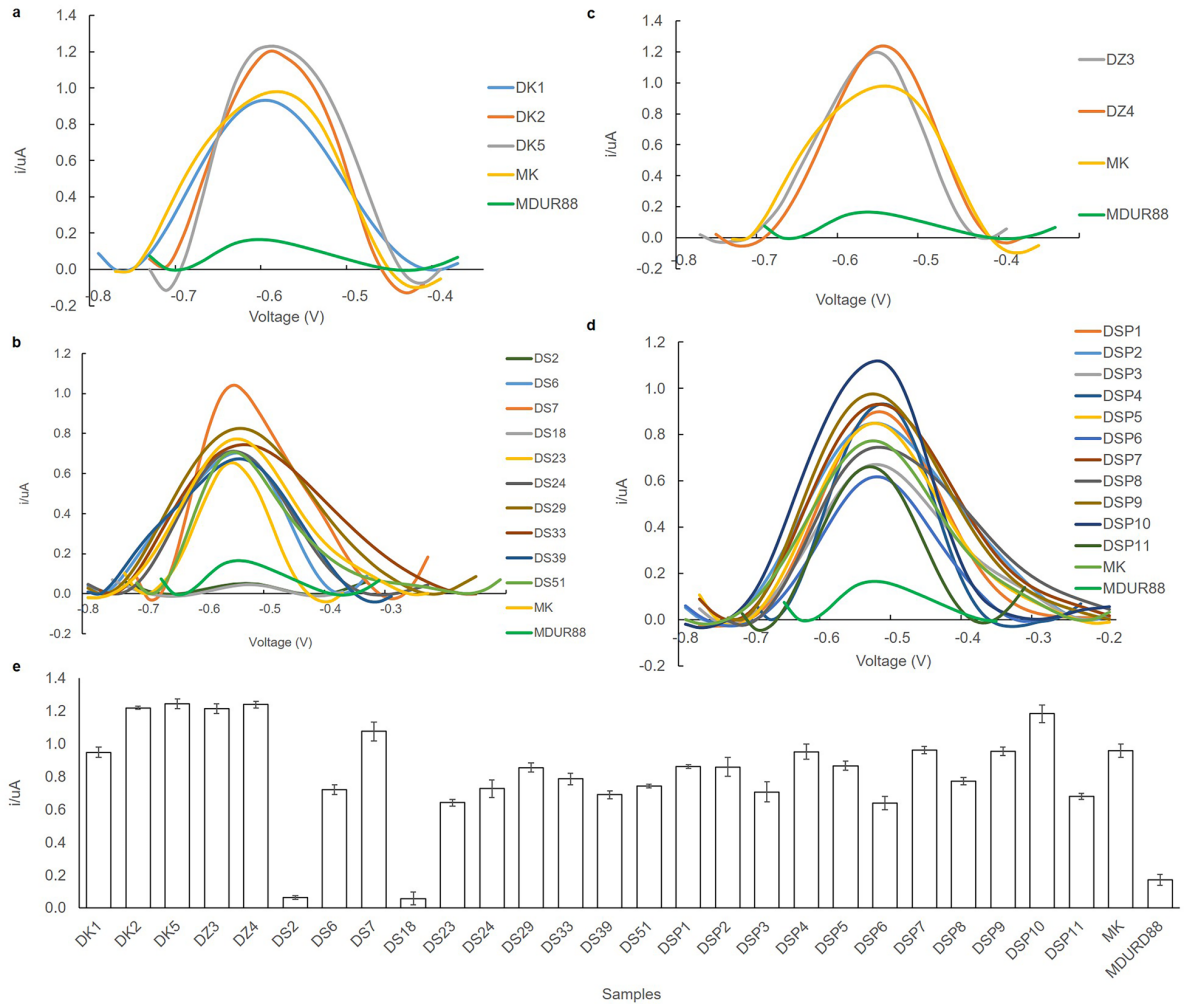
Typical DPV responses of the biosensor to 27 durian field samples which comprised five different locations (DK1 and DK5 from Pulau Raya, Kelantan; DK2 from Batang Merbau, Kelantan (**a**); DS2, DS6, DS23, DS18, DS51, DS59, DS39, DS24, and DS29 from Sintok, Kedah (**b**); DZ3, DZ4, and DZ6 from Zamri Agrofarm, Pahang (**c**); DSP1, DSP2, DSP3, DSP4, SP5, DSP6, DSP7, DSP8, DSP9, and DSP11 from Sungai Petani, Kedah (**d**) and the histogram of the biosensor to the field samples (**e**). MK standard was the positive control, and MDUR88 was the negative control in this study. The DPV peak rate was observed at − 0.55 to − 0.45 V/s versus Ag/AgCl reference electrode in the presence of 10 mM AQMS. Error bars are standard deviations across three repetitive experiments.

In order to prove the effectiveness of the biosensor in determining and distinguishing the Durian MK variety from other Durian varieties, a total of 27 different durian field samples were analysed with the electrochemical DNA biosensor. Figure 5 illustrates the effect of different durian DNA samples on the biosensor response. Based on the DPV diagram in Fig. 5a–d, all field samples were identified as MK variety except for DS2 and DS18 from Sintok, Kedah, which yielded current as low as the non-MK control sample MDUR88. As shown in Fig. 5e, the mean of 24 MK samples is 0.93, and the electrochemical response of the biosensor for all samples was comparable with the MK standard, which revealed the current of 0.93 ± 0.25 µA except for MKS2 and MKS18**.** These results from the electrochemical DNA biosensor have been validated with the standard PCR-based method and followed by sequencing to determine the 27 durian field samples. PCR amplification of 27 durian samples produced 300 bp amplicon except for DS2 and DS18 samples (Fig. S2). With the results tabulated in Table 1, both methods provided the same result for the determination of MK variety. This indicates that the DNA biosensor developed here can be used for accurate determination of MK variety rapidly within 40 min.

**Table 1:** Validation of 27 Durian samples by sequencing of the PCR products in order to identify and validate MK variety from all samples.

No	Sample	Sequence	PCR TEST confirmed Musang King (Yes/No)	Biosenfor Test confirmed Musang King (Yes/No)
1	DZ3	ATTGACGGCTCAAGNACAACCGTATAGAGTTTTGGCTATCCTTTGTATAGATTCC**CATACATGATAGATTCCC**ATACATGTATTGCCAAACCAAACGGGGGATTGAACAAAAAAATGAGTGGATGGTTAGGAACACCAAAAA	Yes	Yes
2	DZ4	TCATTTTGACGGGCTCAAGATCAACCGTATAGAGTTTTGGCTATCCTTTGTATAGATTCC**CATACATGATAGATTCCC**ATACATGTATTGCCAAACCAAACGGGGGATTGAACAAAAAAATGAGTGGATGGTTAGGAACACCAAAT	Yes	Yes
3	DZ6	CAATTGAACGGGCTCAAAGATCAACCGTATAGAGTTTTGGCTATCCTTTGTATAGATTCC**CATACATGATAGATTCCC**ATACATGTATTGCCAAACCAAACGGGGGATTGAACAAAAAAATGAGTGGATGGTTAGGAACACCAAAA	Yes	Yes
4	DK1	CCTATTGAACGGGCTCAAAGAATCAACCGTATAGAGTTTTGGCTATCCTTTGTATAGATTCC**CATACATGATAGATTCCC**ATACATGTATTGCCAAACCAAACGGGGGATTGAACAAAAAAATGAGTGGATGGTTAGGAACACCAAA	Yes	Yes
5	DK5	TTNAGCGGCTCAAAGATCAACCGTATAGAGTTTTGGCTATCCTTTGTATAGATTCC**CATACATGATAGATTCCC**ATACATGTATTGCCAAACCAAACGGGGGATTGAACAAAAAAATGAGTGGATGGTTAGGAACACCAAAA	Yes	Yes
6	DK2	TTGACGGGCTCAAAGNACAACCGTATAGAGTTTTGGCTATCCTTTGTATAGATTCC**CATACATGATAGATTCCC**ATACATGTATTGCCAAACCAAACGGGGGATTGAACAAAAAAATGAGTGGATGGTTAGGAACACCAAAAA	Yes	Yes
7	DSP1	TTGAACGGGCTCAAAGNATCAACCGTATAGAGTTTTGGCTATCCTTTGTATAGATTCC**CATACATGATAGATTCCC**ATACATGTATTGCCAAACCAAACGGGGGATTGAACAAAAAAATGAGTGGATGGTTAGGAACACCAAA	Yes	Yes
8	DSP2	ACGGGCTCAAAGNACAACCGTATAGAGTTTTGGCTATCCTTTGTATAGATTCC**CATACATGATAGATTCCC**ATACATGTATTGCCAAACCAAACGGGGGATTGAACAAAAAAATGAGTGGATGGTTAGGAACACCAAAA	Yes	Yes
9	DSP3	TGTTGACGGGCTCAAGATCAACCGTATAGAGTTTTGGCTATCCTTTGTATAGATTCC**CATACATGATAGATTCCC**ATACATGTATTGCCAAACCAAACGGGGGATTGAACAAAAAAATGAGTGGATGGTTAGGAACACCAAAAA	Yes	Yes
10	DSP4	TCTNTTTGACGGGCTCAAAGATCAACCGTATAGAGTTTTGGCTATCCTTTGTATAGATTCC**CATACATGATAGATTCCC**ATACATGTATTGCCAAACCAAACGGGGGATTGAACAAAAAAATGAGTGGATGGTTAGGAACACCAAAA	Yes	Yes
11	DSP5	CCNATTGACGGGCTCAAAGATCAACCGTATAGAGTTTTGGCTATCCTTTGTATAGATTCC**CATACATGATAGATTCCC**ATACATGTATTGCCAAACCAAACGGGGGATTGAACAAAAAAATGAGTGGATGGTTAGGAACACCAAA	Yes	Yes
12	DSP6	TTTGAACGGCTCAAAGATCAACCGTATAGAGTTTTGGCTATCCTTTGTATAGATTCC**CATACATGATAGATTCCC**ATACATGTATTGCCAAACCAAACGGGGGGATTGAACAAAAAAATGAGTGGATGGTTAGGAACACCAAAAA	Yes	Yes
13	DSP7	ANTGAACGGGCTCAAGAATCAACCGTATAGAGTTTTGGCTATCCTTTGTATAGATTCC**CATACATGATAGATTCCC**ATACATGTATTGCCAAACCAAACGGGGGGATTGAACAAAAAAATGAGTGGATGGTTAGGAACACCAAAAC	Yes	Yes
14	DSP8	AAAATTGAACGGGCTCAAAGNACAACCGTATAGAGTTTTGGCTATCCTTTGTATAGATTCC**CATACATGATAGATTCCC**ATACATGTATTGCCAAACCAAACGGGGGATTGAACAAAAAAATGAGTGGATGGTTAGGAACACCAAAA	Yes	Yes
15	DSP9	TTGACGGCTCAAGATCAACCGTATAGAGTTTTGGCTATCCTTTGTATAGATTCC**CATACATGATAGATTCCC**ATACATGTATTGCCAAACCAAACGGGGGATTGAACAAAAAAATGAGTGGATGGTTAGGAACACCAAAA	Yes	Yes
16	DSP10	TTTTTGACGGCTCAAGATCAACCGTATAGAGTTTTGGCTATCCTTTGTATAGATTCC**CATACATGATAGATTCCC**ATACATGTATTGCCAAACCAAACGGGGGATTGAACAAAAAAATGAGTGGATGGTTAGGAACACCAAAT	Yes	Yes
17	DSP11	TTGAACGGGCTCAANGATCAACCGTATAGAGTTTTGGCTATCCTTTGTATAGATTCC**CATACATGATAGATTCCC**ATACATGTATTGCCAAACCAAACGGGGGATTGAACAAAAAAATGAGTGGATGGTTAGGAACACCAAAA	Yes	Yes
18	DS2	TACNTTTAACGGGCTCAAAGATCAACCGTATAGAGTTTTGGCTATCCTTTGTATAGATTCCCATACATGTATTGCCAAACCAAACGGGGGATTGAACAAAAAAATGAGTGGATGGTTAGGAACACCAAAAA	No	No
19	DS6	TTTGAACGGCTCAAAGATCAACCGTATAGAGTTTTGGCTATCCTTTGTATAGATTCC**CATACATGATAGATTCCC**ATACATGTATTGCCAAACCAAACGGGGGGATTGAACAAAAAAATGAGTGGATGGTTAGGAACACCAAAAA	Yes	Yes
20	DS7	CTATTTGACGGGCTCAAAGATCAACCGTATAGAGTTTTGGCTATCCTTTGTATAGATTCC**CATACATGATAGATTCCC**ATACATGTATTGCCAAACCAAACGGGGGATTGAACAAAAAAATGAGTGGATGGTTAGGAACACCAAATA	Yes	Yes
21	DS18	TGAACGGGCTCAAAGNACAACCGTATAGAGTTTTGGCTATCCTTTGTATAGATTCCCATACATGTATTGCCAAACCAAACGGGGGATTGAACAAAAAAATGAGTGGATGGTTAGGAACACCAAAAT	No	No
22	DS23	NTGAACGGGCTCAAAGAATCAACCGTATAGAGTTTTGGCTATCCTTTGTATAGATTCC**CATACATGATAGATTCCC**ATACATGTATTGCCAAACCAAACGGGGGATTGAACAAAAAAATGAGTGGATGGTTAGGAACACCAAA	Yes	Yes
23	DS24	ANTGAACGGGCTCAAAGATCAACCGTATAGAGTTTTGGCTATCCTTTGTATAGATTCC**CATACATGATAGATTCCC**ATACATGTATTGCCAAACCAAACGGGGGATTGAACAAAAAAATGAGTGGATGGTTAGGAACACCAAAAA	Yes	Yes
24	DS29	ACATTTGACGGGCTCAAAGATCAACCGTATAGAGTTTTGGCTATCCTTTGTATAGATTCC**CATACATGATAGATTCCC**ATACATGTATTGCCAAACCAAACGGGGGATTGAACAAAAAAATGAGTGGATGGTTAGGAACACCAAAAA	Yes	Yes
25	DS33	NTGACGGCTCAAGATCAACCGTATAGAGTTTTGGCTATCCTTTGTATAGATTCC**CATACATGATAGATTCCC**ATACATGTATTGCCAAACCAAACGGGGGATTGAACAAAAAAATGAGTGGATGGTTAGGAACACCAAAA	Yes	Yes
26	DS39	TTGACGGCTCAAANACAACCGTATAGAGTTTTGGCTATCCTTTGTATAGATTCC**CATACATGATAGATTCCC**ATACATGTATTGCCAAACCAAACGGGGGATTGAACAAAAAAATGAGTGGATGGTTAGGAACACCAAAAA	Yes	Yes
27	DS51	CTGTTGACGGGCTCAAAGATCAACCGTATAGAGTTTTGGCTATCCTTTGTATAGATTCC**CATACATGATAGATTCCC**ATACATGTATTGCCAAACCAAACGGGGGATTGAACAAAAAAATGAGTGGATGGTTAGGAACACCAAAAA	Yes	Yes

The bold area contains the unique sequence of MK variety based on *nadhA* gene sequence.

## Conclusion

In this study, an electrochemical DNA biosensor was successfully developed with high specificity, good sensitivity, wide linear response ranges, and low detection limit in the determination of MK durian. Furthermore, the electrochemical DNA biosensor showed a good response to the MK DNA target, which implies that the biosensor is sensitive and has high selectivity to determine MK variety. Moreover, the developed MK DNA biosensor can assist farmers for early identification of MK durian variety in order to avoid confusion in the propagation system, which is economically advantageous in agriculture sectors. Furthermore, farmers can benefit from the use of biosensors for the identification of MK durian variety since it can help them to ensure the authenticity of MK, which is crucial for export activities to foreign nations. Early determination and confirmation of MK’s authenticity at the seedling stage is also critical to ensuring that farmers do not suffer significant losses due to their large investment in Mk durian trees. Employing DNA biosensors to identify MK durian variety also has the advantage of allowing farmers or dealers to obtain proof of certification for export permission to an international market. The identification of MK is also not only beneficial to farmers, but it can also protect the rights of consumers in meeting the high demand for trees or fruit as MK is a durian variety that has a high global market.

## Methods

### DNA probe selection of ‘Musang King’

The ten durian varieties are MK, D99, D145, MDUR88, D175, MDUR78, D24, D168, D200, and MDUR79 maintained at Commercialization and Business Centre, Malaysian Agricultural Research and Development Institute (MARDI). Total DNA was extracted from leaf tissues of ten durian varieties (MDUR88, MK, D24, D99, D145, D168, D175, D200, MDUR78 and MDUR79) (Fig. S3) using DNeasy Plant Pro Kit (Qiagen). Polymerase chain reaction (PCR) amplification of the six candidates DNA barcode (*nadhA, PetB-PetD, trnW-psaJ*, *ITS1*, *matK*, and *rbcL* which were obtained from highly variable chloroplast regions designed by Dong et al.^37^, Cheng et al.^50^, and Teh et al.^11^ (Table 2) was performed in 20 µL reaction mixtures. Each PCR mixture contained 2.0 µL buffer, 2.0 µL dNTPs (2 µmol/L), 1.0 µL each primer (5 µmol/L), 1.0 µL total DNA (25 ng), 0.2 µL *Taq* polymerase (5 µ/mL), and 11.8 µL ddH_2_O. The PCR program was as follows: 94 °C for 3 min, followed by 35 cycles of 94 °C for 30 s, 53 °C for 30 s, 72 °C for 2 min, with a final extension at 72 °C for 5 min. The DNA fragments of PCR product were then separated with 1.5% agarose gel electrophoresis. The DNA sequences of PCR products from ten durian varieties were aligned using Clustal Omega (https://www.ebi.ac.uk/Tools/msa/clustalo/) and unique DNA sequences that can differentiate MK from the other nine varieties were identified and used as a DNA probe in DNA biosensor development for MK determination.

**Table 2:** List of primers used for amplifying ten durian varieties.

No	Locus	Name	Sequence 5’ to 3’	Annealing temperature (°C)	References
1	*nadhA*	nadhA-f	TCAACTATATCAACTGTACTTGAAC	53	Dong et al.^37^
		nadhA-r	CGAGCTGCTGCTCAATCGAT		
2	*PetB-PetD*	petB-f	CAATCCTTTGACTCGTTTT	53	
		petD-r	GGTTCACCAATCATTGATGGTTC		
3	*tnrW-psaJ*	trnW-f	TCTACCGAACTGAACTAAGAGCGC	53	
		psaJ-r	CGATTAATCTCTATCAATAGACCTGC		
4	*rbcL-accD*	rbcL-f	TAGCTGCTGCTTGTGAGGTATGGA	53	
		accD-r	AAATACTAGGCCCACTAAAGG		
5	*matK*	matK-F	ATGGAGGAATTTCAAG	53	Teh et al.^11^
		matK-R	TCA TTC ATG ATT GAC CAG		
6	*ITS1*	ITS-u1	GGAAGKARAAGTCGTAACAAGG	53	Cheng et al.^50^
		ITS-u2	GCGTTCAAAGAYTCGATGRTTC		

### Design of DNA probe for biosensor

DNA probe, target DNA and non-complementary DNA (Table 3) were designed based on the bioinformatics study of *nadhA* gene (based on unique DNA sequence revealed from sequence alignment of 10 durian varieties).

**Table 3:** Oligonucleotides that involved in biosensor study.

Oligonucleotides	Sequences
DNA probe	GGAATCTATCATGTATGGGA(AmC7)
Target DNA	TCCCATACATGATAGATTCC
Non-Com	CTAGGCTTGCACAGTCGAAG

### Instrumentation

Different pulse voltammetry (DPV) experiments were performed with Multi Autolab/M204 with the parameters used were 0.02 V step potential in the scan range of − 0.8 V to − 0.2 V. A carbon screen printed electrode (C-SPE) (Biogenesis Sdn. Bhd.) modified with AcMPs and AuNPs was used as the working electrode. Three paths SPE were used where platinum was the counter electrode and Ag as a reference electrode, and 100 µL of 0.01 M K-phosphate was dropped onto the working electrode. Elma S30H sonicator bath was used to prepare homogeneous solutions.

### Chemicals

2–2-Dimethoxy-2-phenylacetophenone (DMPP), N-acryloxysuccinimide (NAS), anthraquinone-2- sulfonic acid monohydrate sodium salt (AQMS), N-butyl acrylate (n-BA), 1,6-hexanediol diacrylate (HDDA) and colloidal AuNPs were purchased from Sigma-Aldrich. Sodium dodecyl sulphate (SDS) and NaCl were obtained from Systerm. Distilled water was used to prepare all the chemical and biological solutions. The oligonucleotides (Table 1) were purchased from First BASE Laboratories Sdn. Bhd. Oligonucleotide stock solution (100 µM) was diluted with nuclease-free water stored under − 20 °C for further use. Dissolution of oligonucleotide stock solution was performed using 0.05 M K-phosphate buffer pH 7.0. Stock solution of 1.0 mM AQMS was prepared in 0.05 M K-phosphate buffer (pH 7.0) while complementary DNA and non-complementary solutions were prepared with 0.05 M of Na-phosphate buffer at pH 7.0 containing 1.0 mM of AQMS for hybridization current.

### Synthesis of acrylic microsphere

AcMPs were synthesised as reported by Ulianas and team^47^. Briefly, AcMPs were prepared with a mixture of 7 mL of nBA, 0.01 g of SDS, 0.1 g of DMPP, 450 μL of HDDA, 6 mg of NAS and 15 mL of H_2_0 and sonicated at room temperature (25 °C) for 10 min. The resulting emulsion solution was then photocured with UV light for 600 s with ultraviolet radiation of a wavelength ranging from 250 to 350 nm under a continuous flow nitrogen gas. Poly(nBA-NAS) microspheres were then collected by centrifugation at 4,000 rpm for 30 min and subsequently washed in 0.05 M K-phosphate buffer (pH 7.0) for three times, followed by drying at ambient temperature.

### Fabrication of DNA biosensor

**Figure 6:**
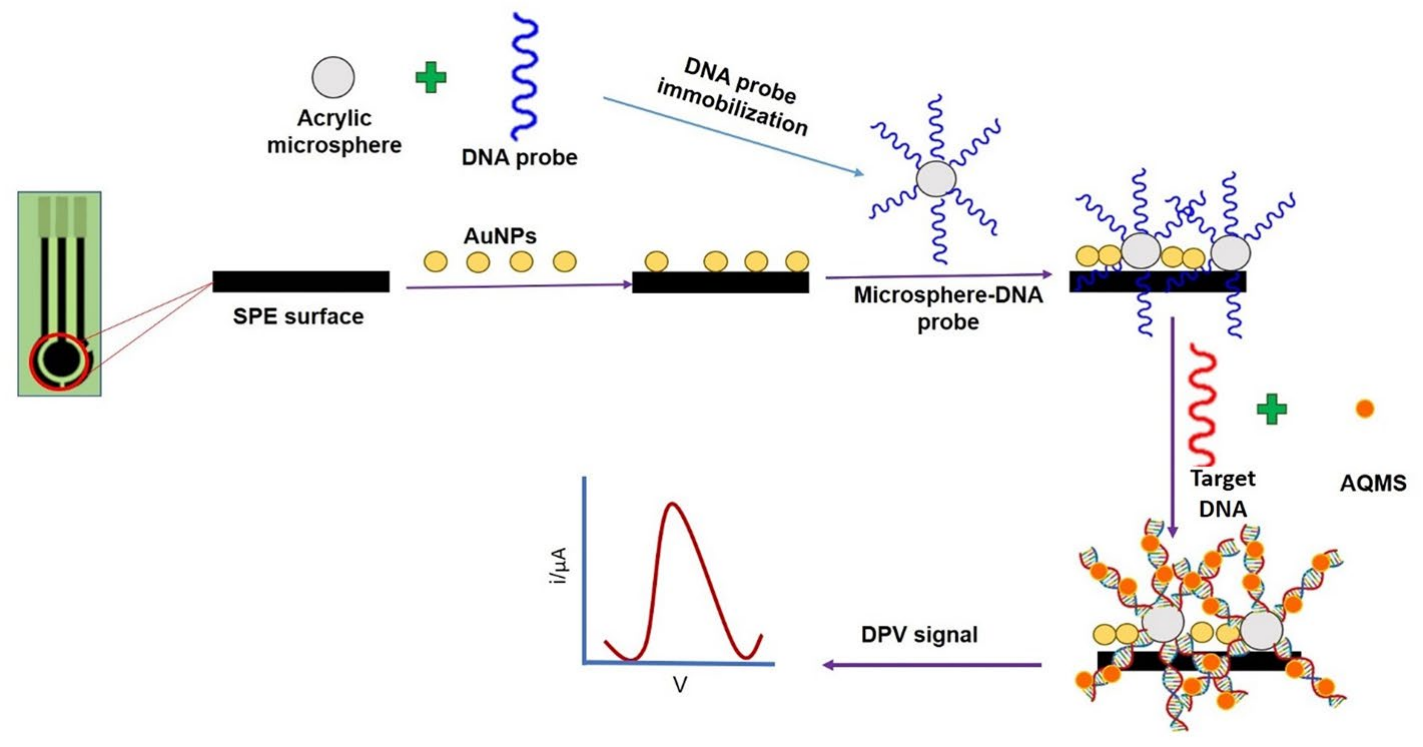
The schematic diagram of electrochemical DNA biosensor based on acrylic microsphere-gold nanoparticle for the determination of MK durian variety.

Two mg of acrylic microspheres were weighed in a microcentrifuge tube and 300 µL of 1 µM animated DNA probes was added to immobilize the DNA onto the microspheres for 24 h at 4 °C. The DNA /AcMPs were collected after 24 h by centrifuging at 1000 rpm for 8 min. The supernatant (which contains DNA probes) was kept for future use in DNA immobilization. The microspheres with K-phosphate were washed several times, and the wash solution was discarded. Fresh 0.05 M K-phosphate (pH 7.0) buffer was added to re-suspense the DNA microspheres. One mg of gold nanoparticles (AuNPs) was suspended in 300 µL ethanol and 10 µL of AuNPs suspension were drop-coated onto C-SPE. The electrode AuNPs/SPE is then left to dry at room temperature for 1–2 h. AcMPs immobilized with DNA probes were pipetted (8 µL) and dropped it onto AuNPs/CSPE and stored at 4 °C until dry for 24 h. 10 µL of solution containing DNA target, 0.05 M of Na-phosphate buffer and 1.0 mM AQMS was dropped onto the electrode surface and incubated for 40 min for hybridization of the probe with the target. The electrode was then rinsed several times with 0.05 M K-phosphate buffer. An amount of 100 µL fresh 0.05 M K-phosphate was dropped onto the electrode surface, and the DPV was scanned at the potential range of (− 0.8 V to − 0.2 V) of the electrode using CSPE as a working electrode, platinum as the counter electrode and Ag as a reference electrode. The AQMS peak should appear at approximately − 0.55 V. All the experiments were performed in triplicate. The SPE construction and design mechanism of the electrochemical DNA biosensor is illustrated in Fig. 6.

### Different concentration of target DNA

The response of DNA biosensor was examined based on the different concentration of target DNA (1.5 µM, 1.0 µM, 0.1 µM, 0.05 µM and 0.01 µM) and no target. All measurements were performed with DPV in a measurement cell containing 0.05 M K-phosphate buffer at pH 7.0 and 10 mM AQMS.

### Specificity of biosensor system

The response of DNA biosensor was examined based on the hybridization effect of genomic DNA from different durian varieties on the immobilized DNA probe on AcMPs. This was performed in the present of ten different durian varieties (MK, D24, MDUR88, MDUR78, MDUR79, D168, D200, D145, D99, D175) and blank (without DNA), in 0.01 M Na-phosphate buffer (pH 7.0) for hybridization of DNA probe.

### Sensitivity of biosensor system

The biosensor experiment was performed on different concentration of MK percentage (100%, 80%, 60%, 40%, 20%, 10%, and 0%), where genomic DNA of MK variety was mixed with genomic DNA from MDUR88 variety. In 1 µL of DNA sample was containing total of 10 ng MK and MDUR88. The combination of MK and MDUR88 gDNA used in this study were shown in the Table 4.

**Table 4:** Combination of MK and MDUR88 gDNA used for the sensitivity test of biosensor system.

Concentration		Final concentration (Molar)	
Musang King	MDUR18	AQMS	Na-phosphate Buffer
0%	100%/10 ng	0.01	0.05
10%/1 ng	90%/9 ng	0.01	0.05
20%/2 ng	80%/8 ng	0.01	0.05
40%/4 ng	60%/6 ng	0.01	0.05
60%/6 ng	40%/4 ng	0.01	0.05
80%/8 ng	20%/2 ng	0.01	0.05
100%/10 ng	0%	0.01	0.05

### Field samples test

A total of 27 durian samples were collected from five different regions in Peninsular Malaysia as shown in Table 5. The optimised DNA biosensor was then used for the determination MK gDNA via DPV method.

**Table 5:** Samples collection of MK from five different locations in Peninsular Malaysia**.**

No	Sample	Sample location
1	DZ3	Zamri Agrofarm, Pahang
2	DZ4	
3	DZ6	
4	DK1	Pulau Raya, Kelantan
5	DK5	Pulau Raya, Kelantan
6	DK2	Batang Merbau, Kelantan
7	DSP1	Sungai Petani, Kedah
8	DSP2	
9	DSP3	
10	DSP4	
11	DSP5	
12	DSP6	
13	DSP7	
14	DSP8	
15	DSP9	
16	DSP10	
17	DSP11	
18	DS2	Sintok, Kedah
19	DS6	
20	DS7	
21	DS18	
22	DS23	
23	DS24	
24	DS29	
25	DS33	
26	DS39	
27	DS51	

### Validation

The biosensor performance of 27 MK samples was validated via PCR based-method using designed primers from *nadhA* gene sequence as shown in Table 6. The reaction mixture and PCR program used in validation were similar as used in the DNA probe selection for MK biosensor development. Amplicons were confirmed using gel electrophoresis. The PCR products were then purified and outsourced for DNA sequencing at 1st BASE, Malaysia. From the DNA sequence of PCR product, unique sequence of MK was identified, thus validate the biosensor performance in identifying MK field samples.

**Table 6:** The primer sequences designed from *nadh*A gene used for the validation of the biosensor performance of 27 MK samples.

Primer name	Primer sequence 5’-3’
DUR-F	TACCCCAAGACGGGTTGAT
DUR-R	TTGGTGTTCCTAACCATCCA

## Supplementary Information

Supplementary Information 1.

Supplementary Information 2.

Supplementary Information 3.

## Data Availability

The datasets generated and analysed during the current study are available in the European Variation Archive (EVA) at EMBL-EBI under accession number PRJEB55857 (https://www.ebi.ac.uk/eva/?eva-study=PRJEB55857). Additional raw data will be available upon request.

## References

[1] Li JX, Schieberle P, Steinhaus M (2012). Characterization of the major odor-active compounds in Thai durian (*Durio zibethinus* L. ‘Monthong’) by aroma extract dilution analysis and headspace gas chromatography-olfactometry. J. Agric. Food Chem..

[2] Siriphanich J (2011). Durian (Durio zibethinus Merr.).

[3] UN Trade Statistics. No Title. https://unstats.un.org.

[4] Morton J (1987). Fruits of Warm Climates.

[5] Department of Agriculture. No Title. http://pvpbkkt.doa.gov.my/ (2019).

[6] Afiq, M., Ariffin, T., Radzuan, S., Razali, N. A. & Sani, M. A. Preliminary study of paternal effect on the characters of ‘Musang king’ Durian (*Durio zibethinus* L.) fruit from cross-pollination. (2019).

[7] Somsri S (2008). Three decades of Durian breeding program in Thailand and its three newly recommended F1 hybrids. Acta Hortic..

[8] Chin ST (2007). Analysis of volatile compounds from Malaysian durians (*Durio zibethinus*) using headspace SPME coupled to fast GC-MS. J. Food Compos. Anal..

[9] Ahmad F, Sugisawa H (2008). Retention Of Volatile Components Of Durian Fruit Leather During Processing And Storage. J. Food Process. Preserv..

[10] Abidin, M. Z., Mohammad, A. G., Shamsudin, M. O., Masdek, N. H. N., & Ghazali, N. M. in *Prosiding Seminar Durian 2000: Kearah Menstabilkan Pengeluaran Kualiti dan Pasaran* 1–3 (2000).

[11] Teh BT (2017). The draft genome of tropical fruit durian (*Durio zibethinus*). Nat. Genet..

[12] Bui TGT, Hoa NTL, Yen JY, Schafleitner R (2017). PCR-based assays for validation of single nucleotide polymorphism markers in rice and mungbean. Hereditas.

[13] Azizi MMF, Lau HY, Abu-Bakar N (2021). Integration of advanced technologies for plant variety and cultivar identification. J. Biosci..

[14] Sales EK (2015). Durian marker kit for durian (*Durio zibethinus* Murr.) identity. World Acad. Sci. Eng. Technol. Int. J. Biol. Biomol. Agric. Food Biotechnol. Eng..

[15] Santoso PJ, Pancoro A, Suhandono S, Aryantha INP (2017). Development of simple-sequence repeats markers from Durian (*Durio zibethinus* Murr. cultv. Matahari) genomic library. Agrivita.

[16] Siew GY (2018). Genetic variation and DNA fingerprinting of durian types in Malaysia using simple sequence repeat (SSR) markers. PeerJ.

[17] Siew GY (2018). Assessment of the genetic variation of Malaysian durian varieties using inter-simple sequence repeat markers and chloroplast DNA sequences. Pertanika J. Trop. Agric. Sci..

[18] Vanijajiva O (2012). The application of ISSR markers in genetic variance detection among Durian (*Durio zibethinus* Murr.) cultivars in the Nonthaburi province, Thailand. Procedia Eng..

[19] Vanijajiva O (2011). Genetic variability among durian (*Durio zibethinus* Murr.) cultivars in the Nonthaburi province, Thailand detected by RAPD analysis. J. Agric. Technol..

[20] Ruwaida IPSP (2009). Variability analysis of Sukun durian plant (*Durio zibethinus*) based on RAPD marker. Nusant. Biosci..

[21] Palumbo F, Barcaccia G (2018). Critical aspects on the use of microsatellite markers for assessing genetic identity of crop plant varieties and authenticity of their food derivatives. Rediscov. Landrac. Resour. Future.

[22] Kirst M, Cordeiro CM, Rezende GDSP, Grattapaglia D (2005). Power of microsatellite markers for fingerprinting and parentage analysis in *Eucalyptus grandis* breeding populations. J. Hered..

[23] Semagn K, Bjørnstad Å, Ndjiondjop MN (2006). An overview of molecular marker methods for plants. Afr. J. Biotechnol..

[24] Drummond TG, Hill MG, Barton JK (2003). Electrochemical DNA sensors. Nat. Biotechnol..

[25] Genes DNA, Kress WJ, Erickson DL (2008). DNA barcodes: Genes, genomics, and bioinformatics. Proc. Natl. Acad. Sci..

[26] Stoeckle MY, Thaler DS (2014). DNA barcoding works in practice but not in (neutral) theory. PLoS ONE.

[27] CBOL Plant Working Group (2009). A DNA barcode for land plants. Proc. Natl. Acad. Sci. U. S. A..

[28] Chen JP, Chiu SH (2000). A poly(N-isopropylacrylamide-co-N-acryloxysuccinimide-co-2-hydroxyethyl methacrylate) composite hydrogel membrane for urease immobilization to enhance urea hydrolysis rate by temperature swing. Enzyme Microb. Technol..

[29] Chaix C, Pacard E, Elaïssari A, Hilaire JF, Pichot C (2003). Surface functionalization of oil-in-water nanoemulsion with a reactive copolymer: Colloidal characterization and peptide immobilization. Colloids Surf. B Biointerfaces.

[30] Rahman M (2017). A highly sensitive electrochemical DNA biosensor from acrylic-gold nano-composite for the determination of arowana fish gender. Nanoscale Res. Lett..

[31] Kleijn SEF, Lai SCS, Koper MTM, Unwin PR (2014). Electrochemistry of nanoparticles. Angew. Chem. Int. Ed..

[32] Riley DJ (2002). Electrochemistry in nanoparticle science. Curr. Opin. Colloid Interface Sci..

[33] Luo X, Morrin A, Killard AJ, Smyth MR (2006). Application of nanoparticles in electrochemical sensors and biosensors. Electroanalysis.

[34] Lin L, Liu Y, Tang L, Li J (2011). Electrochemical DNA sensor by the assembly of graphene and DNA-conjugated gold nanoparticles with silver enhancement strategy. The Analyst.

[35] Liu G (2009). Aptamer-Nanoparticle Strip Biosensor for sensitive detection of cancer cells. Anal. Chem..

[36] Wang W, Fan X, Xu S, Davis JJ, Luo X (2015). Low fouling label-free DNA sensor based on polyethylene glycols decorated with gold nanoparticles for the detection of breast cancer biomarkers. Biosens. Bioelectron..

[37] Dong W, Liu J, Yu J, Wang L, Zhou S (2012). Highly variable chloroplast markers for evaluating plant phylogeny at low taxonomic levels and for DNA barcoding. PLoS ONE.

[38] Rønsted N, Law S, Thornton H, Fay MF, Chase MW (2005). Molecular phylogenetic evidence for the monophyly of *Fritillaria* and *Lilium* (Liliaceae; Liliales) and the infrageneric classification of *Fritillaria*. Mol. Phylogenet. Evol..

[39] Türktaş M, Aslay M, Kaya E, Ertuǧrul F (2012). Molecular characterization of phylogeneticrelationships in *Fritillaria* species inferred from chloroplast trnL-trnF sequences. Turk. J. Biol..

[40] Scarcelli N (2011). A set of 100 chloroplast DNA primer pairs to study population genetics and phylogeny in monocotyledons. PLoS ONE.

[41] Gao YD, Harris A, He XJ (2015). Morphological and ecological divergence of *Lilium* and *Nomocharis* within the Hengduan Mountains and Qinghai-Tibetan Plateau may result from habitat specialization and hybridization. BMC Evol. Biol..

[42] Patterson TB, Givnish TJ (2002). Phylogeny, concerted convergence, and phylogenetic niche conservatism in the core *Liliales*: Insights from rbcL and ndhF sequence data. Evolution (N.Y.).

[43] Bi Y (2018). Chloroplast genomic resources for phylogeny and DNA barcoding: A case study on *Fritillaria*. Sci. Rep..

[44] Xie Q (2019). Morphology and molecular identification of twelve commercial varieties of kiwifruit. Molecules.

[45] Enan M (2018). Cultivar-level phylogeny using chloroplast DNA barcode psbK-psbI spacers for identification of Emirati date palm (*Phoenix dactylifera* L.) varieties. Genet. Mol. Res..

[46] Liu G, Ning H, Ayidaerhan N, Aisa HA (2017). Evaluation of DNA barcode candidates for the discrimination of *Artemisia* L. Mitochondrial DNA Part A DNA Mapp. Seq. Anal..

[47] Ulianas A, Heng LY, Hanifah SA, Ling TL (2012). An electrochemical DNA microbiosensor based on succinimide-modified acrylic microspheres. Sensors (Switzerland).

[48] Batra B, Lata S, Sharma M, Pundir CS (2013). An acrylamide biosensor based on immobilization of hemoglobin onto multiwalled carbon nanotube/copper nanoparticles/polyaniline hybrid film. Anal. Biochem..

[49] Wong ELS, Erohkin P, Gooding JJ (2004). A comparison of cationic and anionic intercalators for the electrochemical transduction of DNA hybridization via long range electron transfer. Electrochem. Commun..

[50] Cheng T (2016). Barcoding the kingdom Plantae: New PCR primers for ITS regions of plants with improved universality and specificity. Mol. Ecol. Resour..

