# Supplementary figures and images for: An electrochemical biosensor for the rapid genetic identification of Musang King durian
Source: Sci Rep. 2022 Nov 11;12:19324. doi: 10.1038/s41598-022-20998-8 (PMC9652400; doi:10.1038/s41598-022-20998-8)

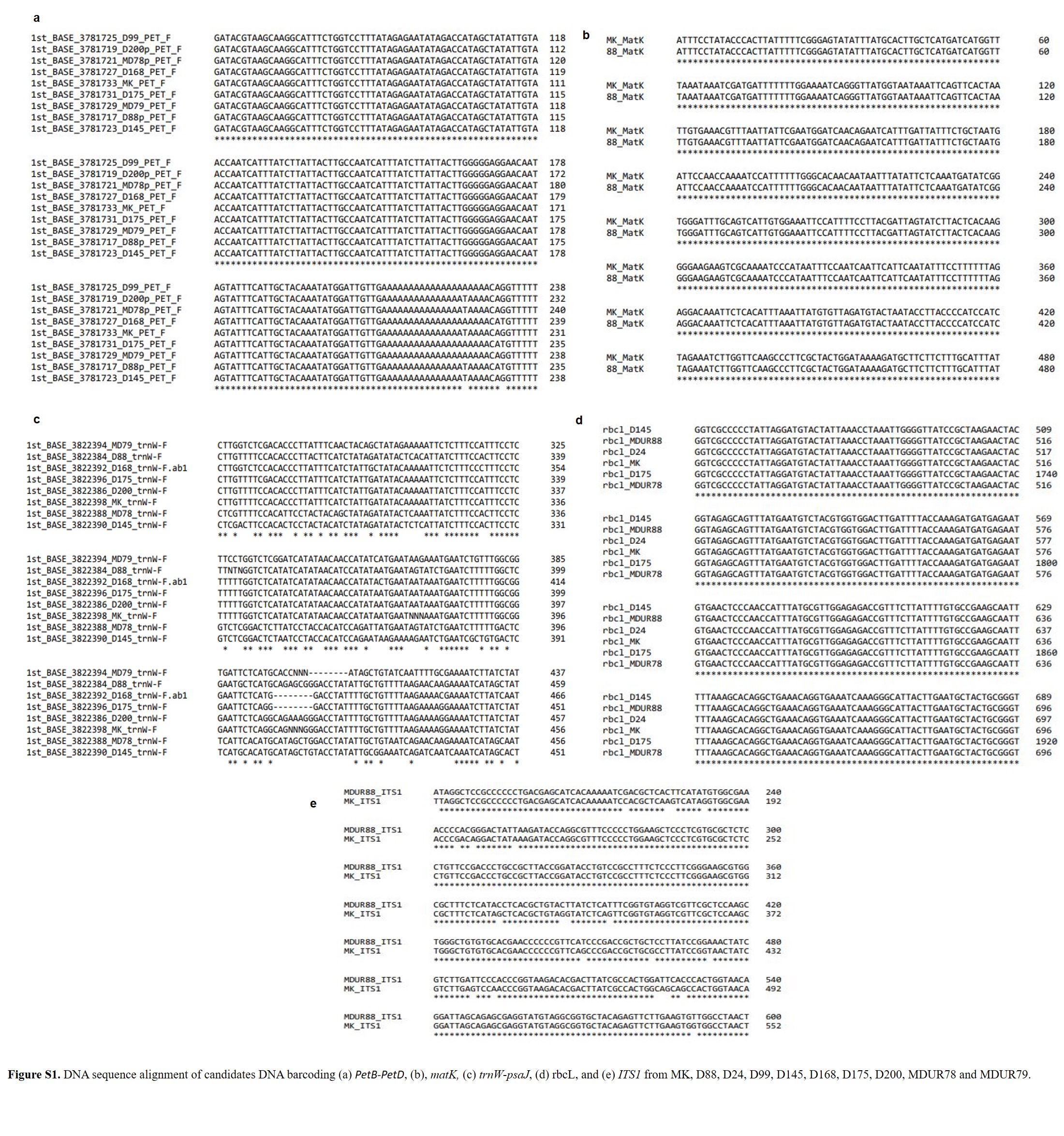

Supplement: Supplementary file 1 — Supplementary Information 1. [file 41598_2022_20998_MOESM1_ESM.jpg]

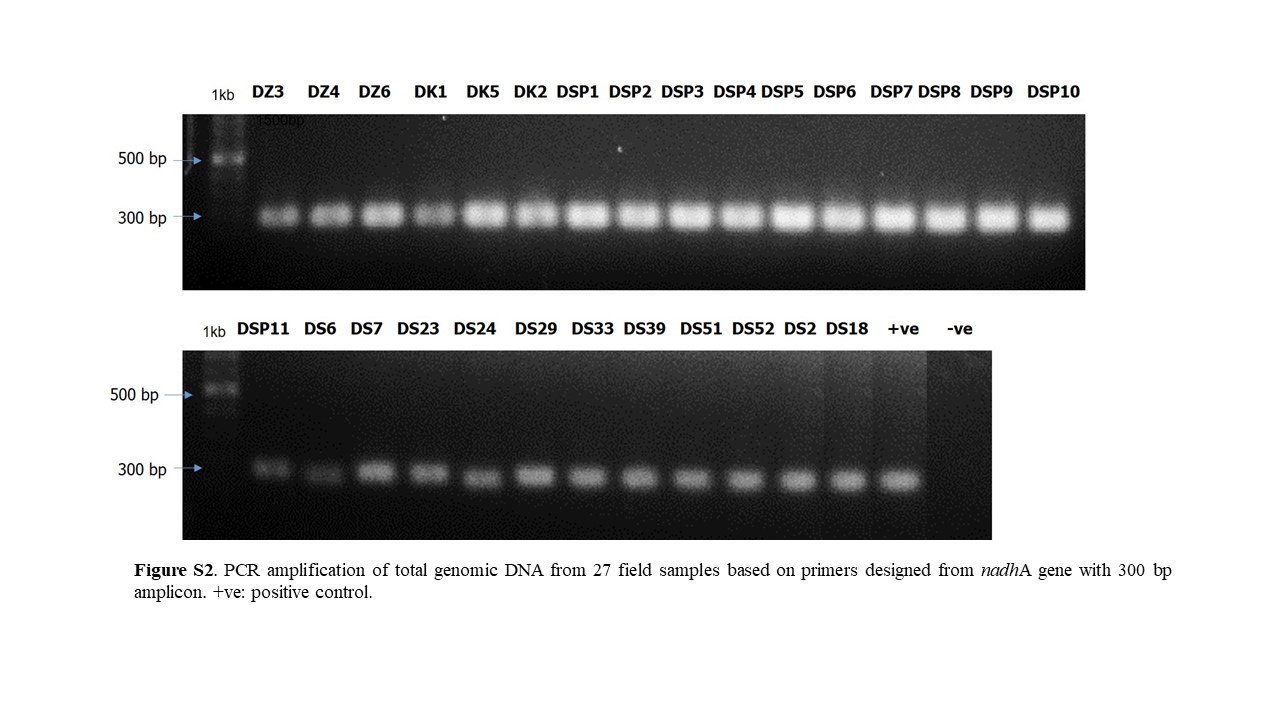

Supplement: Supplementary file 2 — Supplementary Information 2. [file 41598_2022_20998_MOESM2_ESM.jpg]

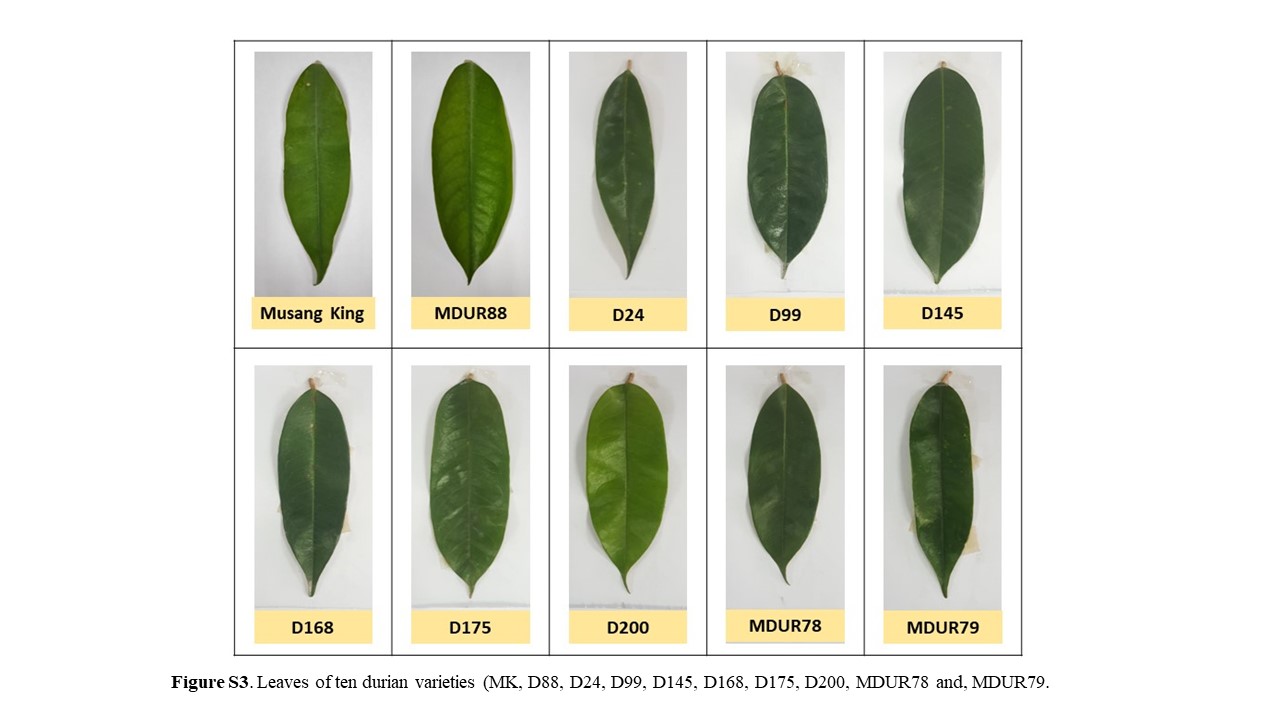

Supplement: Supplementary file 3 — Supplementary Information 3. [file 41598_2022_20998_MOESM3_ESM.jpg]
